# FBXO6 regulates the antiviral immune responses via mediating alveolar macrophages survival

**DOI:** 10.1002/jmv.28203

**Published:** 2022-10-31

**Authors:** Mengyuan Cen, Wei Ouyang, Xiuhui Lin, Xiaohong Du, Huiqun Hu, Huidan Lu, Wanying Zhang, Jingyan Xia, Xiaofeng Qin, Feng Xu

**Affiliations:** ^1^ Department of Infectious Diseases, The Second Affiliated Hospital Zhejiang University School of Medicine Hangzhou China; ^2^ Department of Respiratory Medicine Ningbo First Hospital Ningbo China; ^3^ Institute of Clinical Medicine Research Suzhou Science and Technology Town Hospital Suzhou China; ^4^ Department of Radiation Oncology, The Second Affiliated Hospital Zhejiang University School of Medicine Hangzhou China; ^5^ Institute of Systems Medicine Chinese Academy of Medical Sciences & Peking Union Medical College Beijing China; ^6^ Suzhou Institute of Systems Medicine Suzhou China; ^7^ Research Center for Life Science and Human Health Binjiang Institute of Zhejiang University Hangzhou China

**Keywords:** alveolar macrophages, apoptosis, FBXO6, influenza A, NLRX1

## Abstract

Inducing early apoptosis in alveolar macrophages is one of the strategies influenza A virus (IAV) evolved to subvert host immunity. Correspondingly, the host mitochondrial protein nucleotide‐binding oligomerization domain‐like receptor (NLR)X1 is reported to interact with virus polymerase basic protein 1‐frame 2 (PB1‐F2) accessory protein to counteract virus‐induced apoptosis. Herein, we report that one of the F‐box proteins, FBXO6, promotes proteasomal degradation of NLRX1, and thus facilitates IAV‐induced alveolar macrophages apoptosis and modulates both macrophage survival and type I interferon (IFN) signaling. We observed that FBXO6‐deficient mice infected with IAV exhibited decreased pulmonary viral replication, alleviated inflammatory‐associated pulmonary dysfunction, and less mortality. Analysis of the lungs of IAV‐infected mice revealed markedly reduced leukocyte recruitment but enhanced production of type I IFN in *Fbxo6^−/−^* mice. Furthermore, increased type I IFN production and decreased viral replication were recapitulated in FBXO6 knockdown macrophages and associated with reduced apoptosis. Through gain‐ and loss‐of‐function studies, we found lung resident macrophages but not bone marrow‐derived macrophages play a key role in the differences FBXO6 signaling pathway brings in the antiviral immune response. In further investigation, we identified that FBXO6 interacted with and promoted the proteasomal degradation of NLRX1. Together, our results demonstrate that FBXO6 negatively regulates immunity against IAV infection by enhancing the degradation of NLRX1 and thus impairs the survival of alveolar macrophages and antiviral immunity of the host.

## 1 INTRODUCTION

Influenza A virus (IAV) consists as an enormous threat to public health owing to its panzootic and pandemic potential.^1^ Annually, seasonal influenza virus infections cause serious morbidity and mortality globally, and result in approximately 3,000,000 severe illness cases, which represent a tremendous economic and medical burden.^2^ During the infection, the immune system of the human body has developed various defensive mechanisms to inhibit viral replication. Alveolar macrophages (AMs) are a group of resident macrophages that serve important roles to maintain the homeostasis of alveoli and make quick response to respiratory tract pathogens and particulates. In naive mice, AMs are embryonic derived with unique longevity and capable of self‐renew.^3^, ^4^ Upon IAV infection, AMs are one of the first defensive lines of that interact with the influenza virus by expressing diverse pattern recognition receptors (PRRs) to exert antigen presentation effect.^5^, ^6^ In addition, AMs present potent producers of type I interferons (IFNs) on pulmonary virus infection.^7^, ^8^ Numerous gain and loss of function studies have been carried out to confirm the indispensable role of AMs in influenza infection.^9^, ^10^, ^11^, ^12^, ^13^ On the other hand, viruses have evolved strategies that modulate host responses to facilitate their own survival. It is reported that IAV had the capability to induce the apoptosis of AMs to paralyze its antivirus effect and replicate efficiently.^14^ Recently, several IAV proteins have been identified to be involved in virus‐induced apoptosis, including nucleoprotein,^15^ nonstructural protein 1 (NS1),^16^ neuraminidase,^17^ polymerase basic protein (PB)1‑F2,^18^, ^19^ and matrix protein 1 (M1).^20^ PB1‐F2 was capable of inducing apoptosis in macrophages by targeting mitochondria through its basic amphipathic helix in the C‐terminal region, which could be inhibited by nucleotide‐binding oligomerization domain‐like receptor X1 (NLRX1), a recently recognized member of the NOD‐like receptors. NLRX1 directly interacts with viral protein PB1‐F2 and prevents mitochondrial damage.^19^

Protein ubiquitination is one of the most common regulatory mechanism involved in various biological processes, including signal transduction transcriptional regulation, cell cycle control and apoptosis, and so forth.^19^ When a protein undergoes ubiquitination, a three‐step enzymatic cascade will occur and the last step is a ubiquitin–protein ligase (E3) that recruits both ubiquitin‐bound E2 and the target protein and promotes ubiquitin transfer to the substrate.^21^ F‐box proteins, the critical component of SKP1–Cullin1–F‐box (SCF) family of multisubunit E3 ligases, is identified to be responsible in assembling of SCF E3 complex through interaction with the SKP1 subunit with its highly conserved F‐box domain and determining the substrate specificity for ubiquitination by other less‐conserved and variable parts.^22^, ^23^

FBXO6 is a member of the F‐box protein family initially reported to be involved in endoplasmic reticulum‐related protein degradation through ubiquitination of several glycoproteins by its FBA domain.^24^, ^25^, ^26^ Besides glycoproteins, ubiquitination and degradation of other proteins named Chk1 and RIOK1 are also mediated by FBXO6 according to previous findings.^27^, ^28^ Moreover, recent studies have found FBXO6 exerted controversial functions in the progress of cancer.^29^, ^30^, ^31^ Our previous work also found FBXO6 was associated with type I IFN production regulation, where we elucidated FBXO6 interacted with IFN‐regulatory factor 3 (IRF3) via its FBA domain and promoted ubiquitinated degradation of IRF3 by a noncanonical mechanism.^32^ In the present study, we report that FBXO6 exerts a detrimental function during IAV infection by promoting the virus‐induced apoptosis of AMs via an NLRX1‐dependent mechanism.

## 2 MATERIALS AND METHODS

### 2.1 Mice

The animal experiments were conducted with the approval of the Ethics Committee of the Second Affiliated Hospital of Zhejiang University, and all the experimental protocols were in accordance with the National Institutes of Health Guide for the Care and Use of Laboratory Animals. Specific pathogen free C57BL/6 female mice (6–8 weeks old) were purchased from the Animal Center of Slaccas. *Fbxo6^−/−^* mice were obtained from BRL medicine.

### 2.2 Bone marrow (BM) transplantation

BM transplantation was processed according to the previous studies.^33^ Wild‐type (WT) mice were exposed to irradiation of a dose of 7‐Gy with lead strips shielded their lungs to protect resident AMs. Six hours after irradiation, 1 × 10^7^ congenic BM cells derived from WT and *Fbxo6^−/−^* mice were adoptively transferred via tail vein injection to replenish the BM of irradiated mice. Eight weeks following BM‐reconstitution, circulating blood cells were collected and evaluated using an FBXO6 genotyping analysis.

### 2.3 AMs depletion

Mice were injected intratracheal with 50 μl clodronate liposomes (Liposoma) to deplete AMs 2 days before the virus challenge. Control mice received an equal volume of empty control liposomes. AMs depletion was verified by cell flow cytometry using an ACEA NovoCyteTM (ACEA Biosciences).

### 2.4 Lung histology

Lungs from untreated and virus‐infected mice were fixed with a 4% paraformaldehyde neutral buffer solution and embedded with paraffin. Then 4‐mm sections were stained with hematoxylin and eosin and examined by photo‐microscope (Olympus).

### 2.5 Cell counting in bronchoalveolar lavage fluid (BALF)

BALF was collected and centrifuged for 10 min at ×3000*g*. The erythrocytes of the cell pellets were removed using a lysis buffer. The total cell was counted in the counting plate. Then cells were smeared on a slide and stained with Giemsa reagent for cell differentiation.^34^

### 2.6 Quantitative real‐time polymerase chain reaction (PCR)

Total cellular RNA extraction was performed with an ultrapure RNA kit (Cwbiotech) according to the manufacturer's protocol. Complementary DNA Synthesis was performed using a cDNA synthesis system (Takara). cDNA amplification was conducted with an SYBR Green PCR Kit (Takara) in a CFX96 Touch Real‐Time PCR Detection System (Bio‐Rad). β‐actin served as the internal reference. The comparative Ct method (2^−ΔΔCt^) was used to analyze data.^35^ The sequences of primers for quantitative PCR (qPCR) were displayed in Supporting Information.

### 2.7 Cells and virus

Madin Darby canine kidney cells (MDCKs), MH‐S, and HEK293T cells were obtained from American Type Culture Collection, and cultured in RPMI 1640 or DMEM medium supplemented with 10% fetal bovine serum, 100 U/ml of penicillin and 100 μg/ml of streptomycin at 37°C in a 5% CO_2_ atmosphere.

Influenza virus strain A/PR/8/34 and PR8‐Renilla were grown in MDCKs at a multiplicity of infection (MOI) of 0.1 with 2 μg/ml TPCK trypsin. The virus was harvested by cycles of freeze and thaw, and then centrifugated at 5000 × *g* for 20 min to remove cell debris. The supernatant was subpackaged and stored at −80°C. Viral titers were measured by 50% tissue infectious dose (TCID50) assay in MDCK cells, according to the Reed‐Muench method.

### 2.8 Co‐immunoprecipitation (Co‐IP), Western Blot, and immunofluorescence

For Co‐IP analysis, whole cells were lysed in Lysis Buffer for WB/IP assays (Yeasen) containing a protease inhibitor cocktail, followed by incubation with specific antibodies and protein A/G beads (MedChemExpress) overnight. The beads were washed with 1%‐Triton buffer and eluted with 1× SDS Loading Buffer.

For Western Blot analysis, cells were lysed in RIPA Lysis Buffer (Yeasen) with 1 mM phenylmethylsulfonyl fluoride and protease inhibitor cocktail. Protein concentrations were determined by the BCA Protein Assay Kit (Beyotime). Equal amounts of proteins were loaded and separated on an SDS‐PAGE gel, transferred onto a PVDF membrane (Millipore), and combined with appropriate antibodies for chemiluminescence system (New Cell & Molecular Biotech Co., Ltd.) detection.

Four percent of paraformaldehyde was used to fix tissues or cells, and after 15 min, samples were permeabilized with 0.5% Triton X‐100 for 20 min and then blocked with 4% goat serum for 30 min. After that, samples were incubated with appropriate primary antibodies (1:100) at 4°C overnight and proceeded to secondary antibody (1:2000) incubation. Cells and tissues were photographed under an FV3000 fluorescent microscope (Olympus).

### 2.9 Antibodies and reagents

Primary antibodies against His‐tag, Flag‐tag, and Myc‐tag were purchased from Beyotime Biotechnology. Primary antibodies against β‐Actin and NLRX1 were purchased from Huaan biotechology and Cell Signaling Technology. Antibodies against ki‐67, F4/80, CD11b, and CD11c were obtained from BD. FITC Annexin V apotosis detection kit were purchased from BD. Emricasan was obtained from Selleck. Protein A/G Magnetic Beads (HY‐K0202), MG‐132 (HY‐13259), cycloheximide (CHX) (HY‐12320), and Chloroquine (HY‐17589) were purchased from MedChemExpress.

### 2.10 ELISA

Concentrations of IFN‐α and IFN‐β were measured by ELISA kits (Elabscience Biotechnology Co., Ltd.) according to the manufacturer's instructions.

### 2.11 Flow cytometric analysis

Amounts, proliferation, and apoptosis of alveolar macrophages were analyzed by flow cytometry. The procedure was performed according to the previous description.^35^ Briefly, the cells were digested, washed twice with cold PBS, and resuspended in the binding buffer in a concentration of 1 × 10^6^ cells/ml. Then cells were stained with FITC Annexin V (5 µl), propidium iodide (5 µl) or spesific antibodies (5 µl)per sample for 15 min in the dark at room temperature. Finally, cellular fluorescence was analyzed using an ACEA NovoCyteTM (ACEA Biosciences).

### 2.12 Small interfering RNA (siRNA) transfection

siRNA transfection was conducted using Hieff Trans™ Liposomal Transfection Reagent (Yeasen Biotechnology Co., Ltd.) in accordance with the manufacturer's instructions. All of the siRNAs were designed and synthesized by GenePharma. The siRNA sequences targeting mouse FBXO6 were as follows: sense, 5′‐CCCACACCUUCUCUGAUUATT‐3′, and antisense, 5′‐UAAUCAGAGAAGGUGUGGGTT‐3′. The siRNA sequences targeting mouse NLRX1 were: sense, 5′‐GCUUUCUACGCCUGAACUUTT‐3′, and antisense: 5′‐AAGUUCAGGCGUAGAAAGCTT‐3′. The scramble control sequences were: sense, 5′‐UUCUCCGAACGUGUCACGUTT‐3′, and antisense: 5′‐ACGUGACACGUUCGGAGAATT‐3′.

### 2.13 Statistical analysis

All the statistical analysis was performed with prism 8 (GraphPad). Data are displayed as mean ± SEM. The differences between two groups were analyzed using Student's *t*‐test. Survival curve analysis was evaluated by the log‐rank test. A difference was considered statistically significant if *p* < 0.05.

## 3 RESULTS

### 3.1 FBXO6 expression is elevated in patients infected with IAV

**Figure 1:**
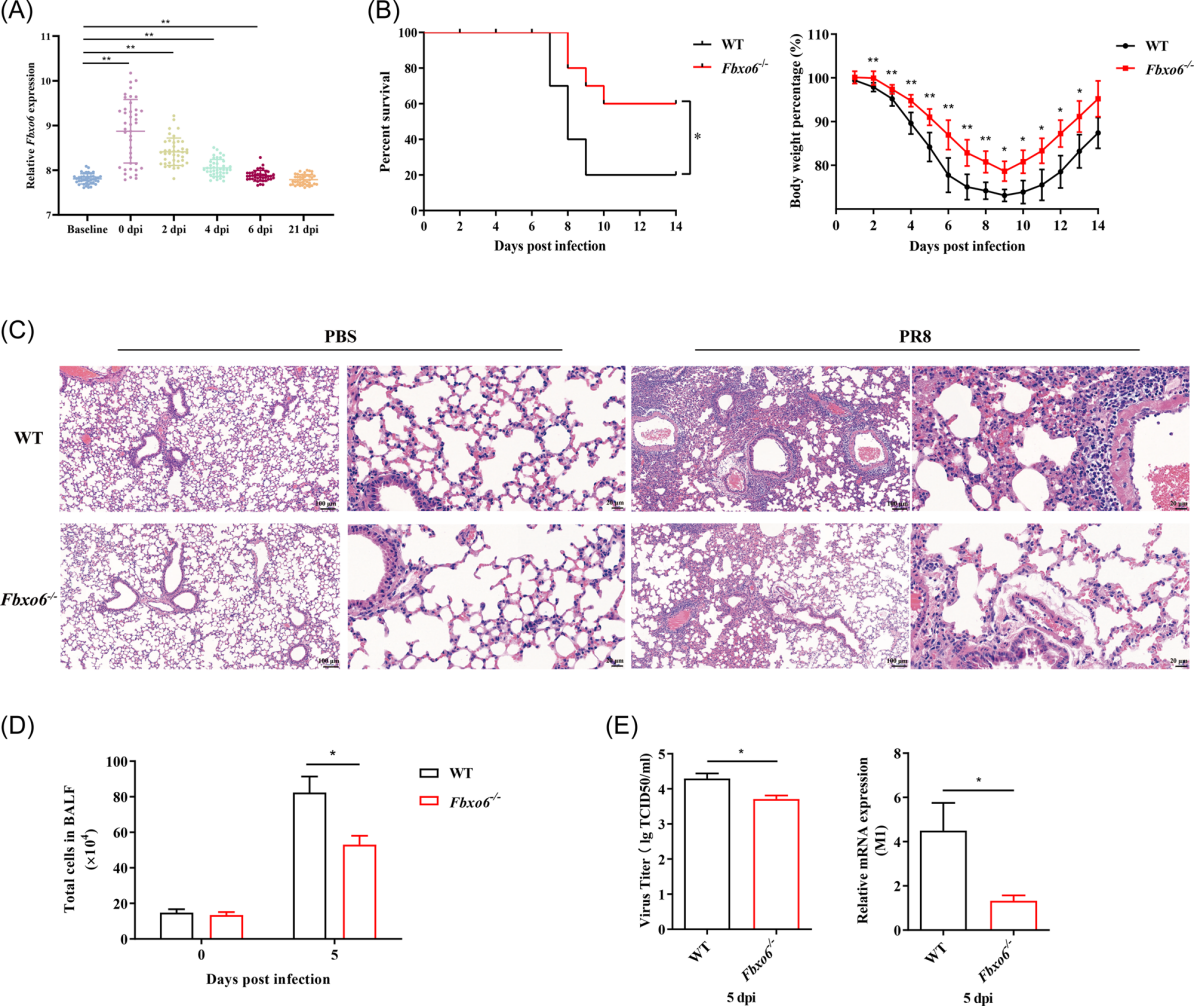
FBXO6 expression is elevated in patients infected with IAV and FBXO6 deficiency enhances antiviral responses in vivo. (A) GEO data sets (GSE68310) were searched and analyzed. (B) Survival and body weight in WT or *Fbxo6^−/−^* mice were monitored after infected with 10^3^ TCID50 PR8 intranasally (*n* = 10 per group). (C–E) WT or *Fbxo6* *^−/−^* mice were challenged intranasally with 10^3^ TCID50 PR8 for 5 days. H&E staining of lung sections was observed. Magnification, ×100 or ×400 (C). The number of total cells was counted (D). Virus titer and the expression of M1 in lungs were determined (E). Quantitative data were presented as mean ± SEM, *p < 0.05; **p < 0.01. H&E, hematoxylin and eosin.

To determine what role FBXO6 plays during IAV infection, we searched GEO data sets (GSE68310) about related research and found that compared with the baseline of the participants, peripheral blood (PB) leukocytes from samples infected with IAV showed a significant increase of expression of *FBXO6* in the first 6 days during the course of infection. As the days passed by, the *FBXO6* expression decreased gradually until the 21st day, the expressions showed no difference between the baseline and postinfection groups (Figure 1A).

### 3.2 FBXO6 deficiency enhances antiviral responses in vivo

To gain insight into the functional significance of FBXO6 in host antiviral immune response in vivo, we challenged *Fbxo6^−/−^* mice intranasally with PR8, and found FBXO6 deficiency decreased morbidity as shown by reduced weight loss and mortality as well (Figure 1B). Additionally, compared with WT mice, *Fbxo6^−/−^* mice showed a noticeably lower level of leukocyte infiltration and alveolar wall thickening by histopathological analysis at 5 days postinfection (dpi) (Figure 1C). To quantify the histological observation, we counted the number of total cells in BALF and found it was much higher in WT than in *Fbxo6^−/−^* mice (Figure 1D). Considering that massive viral replication accompanied by early robust immune responses are major determining factors of the severity of pneumonia, we then measured M1 expression and titer of the virus in lungs from both *Fbxo6^−/−^* and WT mice to detect the replication of PR8 in lungs of the mice and discovered a higher replication of PR8 in WT mice (Figure 1E). These data suggested that *Fbxo6^−/−^* mice represented stronger control against viral infection than WT mice.

### 3.3 FBXO6‐deficient mice have an enhanced type I IFN response to IAV infection

**Figure 2:**
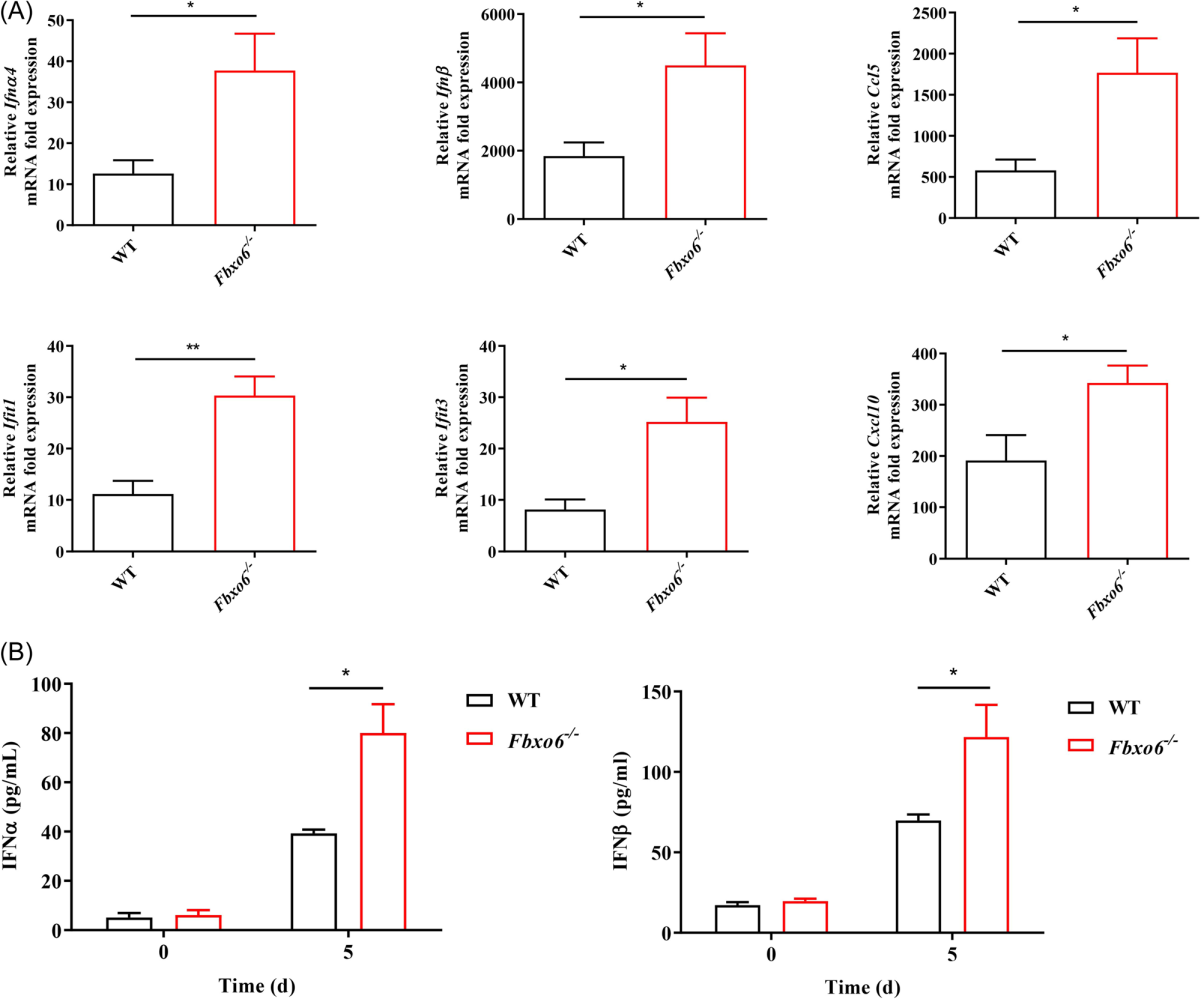
FBXO6‐deficient mice have an enhanced type I IFN response to IAV infection. (A‐B) WT or *Fbxo6^−/−^* mice were challenged intranasally with 10^3^ TCID50 PR8 for 5 days. *n* = 3–6 per group. The expression of *Ifnα4, Ifnβ*, *Ifit1*, *Ifit3*, *Ccl5*, and *Cxcl10* in lung was determined by qPCR (A). The cytokine production of IFN‐α and IFN‐β in BALF was measured by ELISA (B). Quantitative data were presented as mean ± SEM, *p < 0.05; **p < 0.01. BALF, bronchoalveolar lavage fluid; IFN, interferon; qPCR, quantitative polymerase chain reaction.

Upon infection, IAV will actively suppress the type I IFN production in the host to promote replication.^36^ Therefore, the capability of the host to resist this suppression through inducing a strong and swift type I IFN response is particularly critical to limit extensive early viral replication. To determine whether FBXO6 deficiency would alter the type I IFN response of mice following IAV infection, we detected type I IFN mRNA transcription in the lungs and found a significant increase of type I IFN expression in *Fbxo6^−/−^* comparison with WT mice, associating with a notable upregulation in mRNA transcripts of *Ifnα4*, *Ifnβ* and IFN‐stimulated genes (ISGs), including *Ifit1*, *Ifit3*, *Ccl5* and *Cxcl10* (Figure 2A). In the meantime, type I IFN‐α and ‐β protein secretion was also enhanced in the BALF of *Fbxo6^−/−^* mice (Figure 2B). Thus, consistent with the decreased viral replication during IAV infection, deficiency in FBXO6 leads to an extensively enhanced type I IFN response correlated with upregulated ISGs expression in the lungs.

### 3.4 FBXO6 deficiency protects AMs from apoptosis induced by IAV

**Figure 3:**
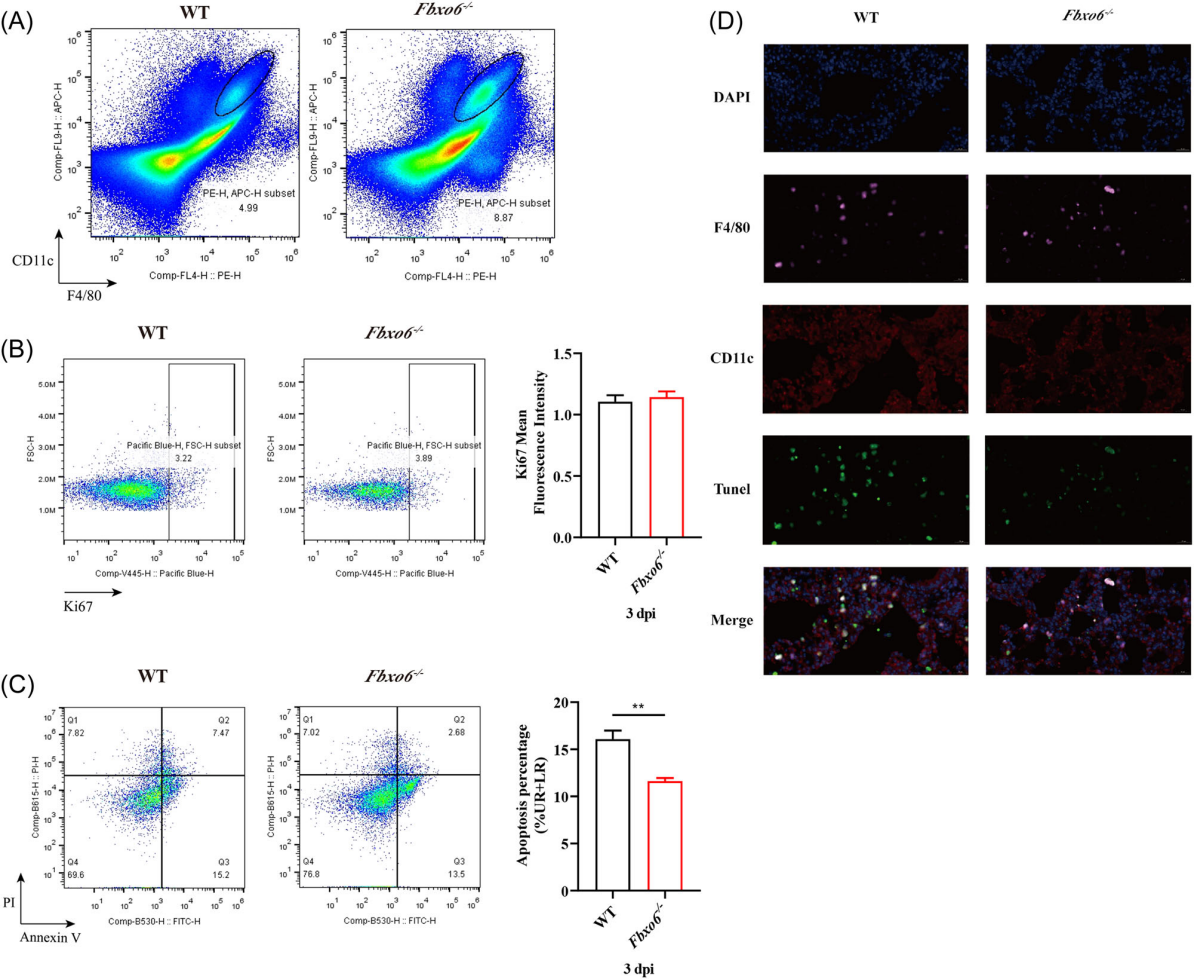
FBXO6 deficiency protects AMs from apoptosis induced by IAV. (A–C) WT or *Fbxo6^−/−^* mice were challenged intranasally with 10^3^ TCID50 PR8 for 3 days , and then amounts (A), proliferation (B), and apoptosis (C) of alveolar macrophages were analyzed by flow cytometry. (D) WT or *F* *b* *x* *o* *6* *^−/−^* mice were challenged intranasally with 10^3^ TCID50 PR8 for 3 days and then Tunel, CD11c, F4/80, and DAPI were detected by immunofluorescence (magnification, ×600). Quantitative data were pbresented as mean ± SEM, *p < 0.05; **p < 0.01. AM, alveolar macrophage; IAV, influenza A virus.

AMs have been reported to play an essential role at the early stage of viral infection, especially in the first 3 dpi. To explore the change of AMs in this progress, we performed a flow cytometric analysis to measure the frequency of AMs in the lungs at 3 dpi of PR8. As shown in Figure 3A, the proportion of AMs was remarkably increased in *Fbxo6^−/−^* mice. To further investigate the reason of the decrease of AMs in WT mice, we detected both the proliferation and apoptosis frequencies of AMs in the lungs of *Fbxo6^−/−^* and WT mice after infection with PR8 for 3 days. Figure 3B,C suggested that the proliferation proportions of AMs in *Fbxo6^−/−^* and WT mice exhibited no significant difference while the frequencies of apoptosis revealed drastic variations. The ratio of apoptosis AMs was much lower in *Fbxo6^−/−^* mice in comparison with WT mice (Figure 3D). Collectively, these results suggest that FBXO6 is detrimental to the survival of AMs in IAV infection which may be attributed to the additional apoptosis of AMs.

### 3.5 Resident AMs not BM‐derived recruited cells play the dominant part in the antiviral immunity upon FBXO6 deficiency

**Figure 4:**
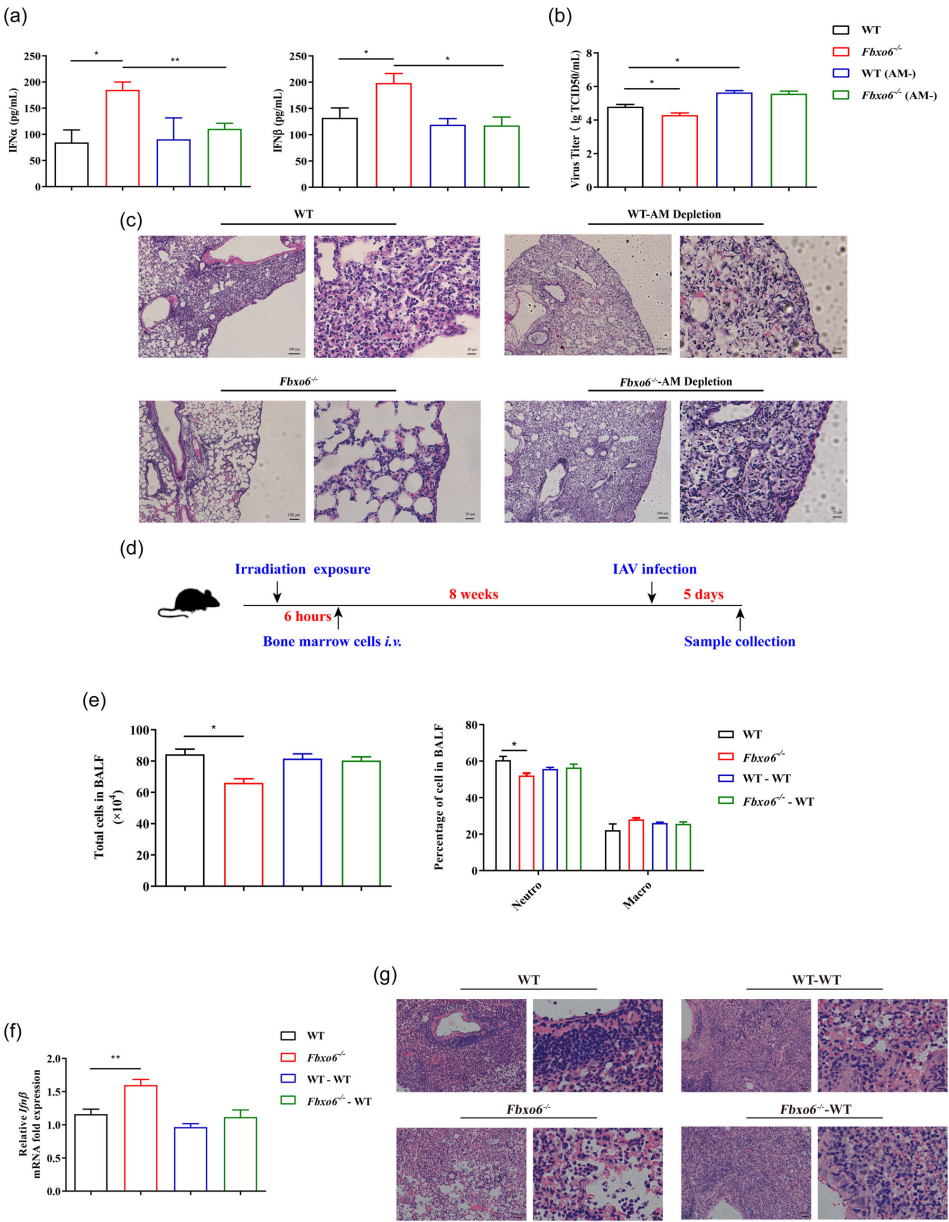
Resident alveolar macrophages, not bone marrow (BM)‐derived recruited cells, play the dominant part in the antiviral immunity upon FBXO6 knockout. (A–C) WT or *Fbxo6^−/−^* mice were treated with 50 μl clodronate liposomes for 2 days before they were challenged intranasally with 10^3^ TCID50 PR8 for 5 days, then IFN‐α and IFN‐β in BALF (A), virus titers in lungs (B), and H&E staining of lung sections (C) were determined. Magnification, ×100 or ×400. (D–G) Wild‐type recipient mice were lethally irradiated (7‐Gy) and reconstituted with BM from WT or *Fbxo6^−/−^* donor mice (WT–WT and *Fbxo6^−/−^*‐WT, respectively). After 8 weeks, BM reconstituted mice and nonirradiated/nonreconstituted *Fbxo6^−/−^* and WT control mice were challenged intranasally with 10^3^ TCID50 PR8 for 5 days (D). The number of total cells and cell classification were measured in BALF (E). The expression of *Ifnβ* in lungs was detected by qPCR (F). H&E staining of lung sections was observed; magnification, ×100 or ×400 (G). Quantitative data were presented as mean ± SEM, *p < 0.05; **p < 0.01. BALF, bronchoalveolar lavage fluid; H&E, hematoxylin and eosin; IFN, interferon; qPCR, quantitative polymerase chain reaction.

Since type I IFN resources in the lung are mainly comprised of both resident macrophages and recruited BM‐derived macrophages, we wanted to determine which population of macrophages played the dominant part in the viral defense upon FBXO6 knockout. Thus, we conducted AMs depletion in both *Fbxo6^−/−^* and WT mice. AMs depletion was confirmed by flow cytometric analysis (Supporting Information: Figure S1A). Then we detected the concentration of IFN‐α and IFN‐β in BALF at 5 dpi. Interestingly, FBXO6‐deficient mice had significantly increased levels of secreted IFN‐α and IFN‐β than WT mice, but the mice depleted of AMs from both genotypes showed no difference in IFN‐α and IFN‐β secretion (Figure 4A). Correspondingly, viral titers in the lung of FBXO6‐deficient mice at 5 dpi were remarkably lower compared with WT mice, but the difference above was not observed in AMs depletion groups (Figure 4B). Consistent with the results in Figure 1C, the histopathological analysis demonstrated that *Fbxo6^−/−^* mice had less inflammatory cells infiltration and decreased alveolar wall thickening at day 5 post PR8 infection than WT mice. However, mice depleted of AMs all exhibited severe lung tissue inflammation injury (Figure 4C). To confirm the role AMs played in different antiviral response in *Fbxo6^−/−^* and WT mice, we performed BM reconstitution. WT mice firstly received lethal irradiation and were then reconstituted with BM from donor WT mice or FBXO6‐deficient mice to create two groups: (1) WT mice reconstituted with WT BM (WT BM/WT mice) and (2) WT mice reconstituted with FBXO6‐deficient BM (*Fbxo6^−/^^−^* BM/WT mice). After 8 weeks post reconstitution, these BM‐reconstituted mice and normal nonirradiated/nonreconstituted WT and FBXO6‐deficient mice were infected intranasally with PR8. At 5 dpi, total cell numbers and cells stained for differential cell identification were determined in BALF collected from mice, and we found that total cell numbers and the proportion of neutrophils were significantly reduced in *Fbxo6^−/−^* mice than WT mice. But in the two BM reconstituted groups, these indications showed no difference (Figure 4E). Furthermore, IFN‐β expression was quantified in the lung tissue following PR8 infection in the four groups. As shown in Figure 4F, IFN‐β transcription was significantly elevated in *Fbxo6^−/−^* mice in comparison with WT mice. However, in FBXO6‐deficient BM reconstituted WT mice, the expression of IFN‐β did not show any difference with WT BM reconstituted mice. Hematoxylin and eosin (H&E) staining also illustrated reduced inflammatory injury in *Fbxo6^−/−^* mice than WT mice, but not BM reconstituted mice (Figure 4G). Taking all these results into consideration, we speculate that phenotypic changes in FBXO6‐deficient mice may be dependent upon the alteration of the amounts and function of AMs.

### 3.6 FBXO6 negatively regulates antiviral immune response and induces apoptosis of AMs in vitro

**Figure 5:**
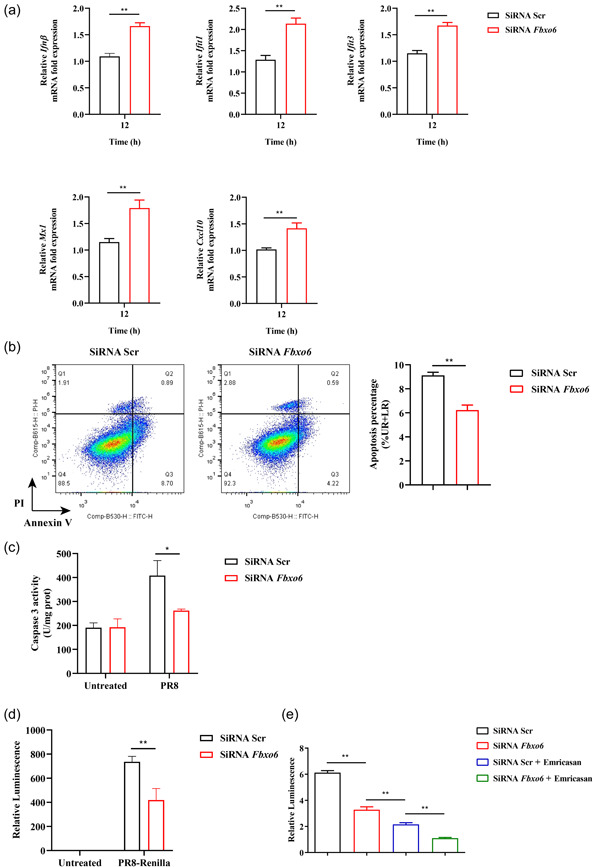
FBXO6 induces apoptosis of alveolar macrophages in vitro. (A) and (B) MH‐S were transfected with *Fbxo6* or scramble siRNA 24 h before stimulated with PR8 (MOI = 1), after 12 h, the expression of *Ifnβ*, *Ifit1*, *Ifit3*, *Mx1*, and *Cxcl10* was determined by qPCR (A), and after 24 h, apoptosis of MH‐S was analyzed by flow cytometry (B) and activity of caspase 3 was detected (C). (D) MH‐S cells were transfected with *Fbxo6* or scramble siRNA for 24 h and then infected with PR8‐Renilla (MOI = 1) for 24 h. Then luminescence was measured. (E) MH‐S cells were transfected with *Fbxo6* or scramble siRNA for 24 h and then infected with PR8‐Renilla (MOI = 1) and treated with or without emricasan (10 μM) for 24 h. Quantitative data were presented as mean ± SEM, *p < 0.05; **p < 0.01. MOI, multiplicity of infection; qPCR, quantitative polymerase chain reaction; siRNA, small interfering RNA.

Then we addressed whether there were similar results in vitro, we used MH‐S, a mouse AM cell line, to confirm these findings. Firstly, we utilized small interfering RNA to knockdown the expression of FBXO6 in MH‐S, and then infected both the knockdown and control group of MH‐S with PR8 (MOI = 1). Twelve hours postinfection, the expression of IFN‐β and some ISGs, including *Ifit1*, *Ifit3*, *Mx1*, and *Cxcl10* were significantly upregulated in FBXO6 knockdown MH‐S compared with control (Figure 5A). In addition, we determined the apoptosis and activity of caspase 3 in FBXO6 knockdown and control group of MH‐S and found the apoptosis and activity of caspase 3 were also inhibited by FBXO6 knockdown (Figure 5B,C). Furthermore, to investigate the ability to control viral replication, we stimulated MH‐S knocked down of FBXO6 and control with PR8‐Renilla and the result revealed decreased replication of the virus in FBXO6 knockdown MH‐S (Figure 5D). To address what role FBXO6‐deficiency against PR8‐induced apoptosis plays in this progress, we used a pan caspase inhibitor emricasan to suppress the apoptosis of AMs, and detected the viral burden. We found that in consistent with Figure 5D, the relative luminescence in the FBXO6 knockdown group was reduced compared to the control group. But when apoptosis of AMs was inhibited, a more appreciable decrease was also observed in the control group treated with emricasan than the FBXO6 knockdown group. In the FBXO6 knockdown group treated with emricasan group, the relative luminescence was even lower than the control group treated with emricasan (Figure 5E). These results suggested that cell death mediated by FBXO6 negatively regulated the antiviral immune response in AMs.

### 3.7 FBXO6 interacts with and inhibits the expression of NLRX1

**Figure 6:**
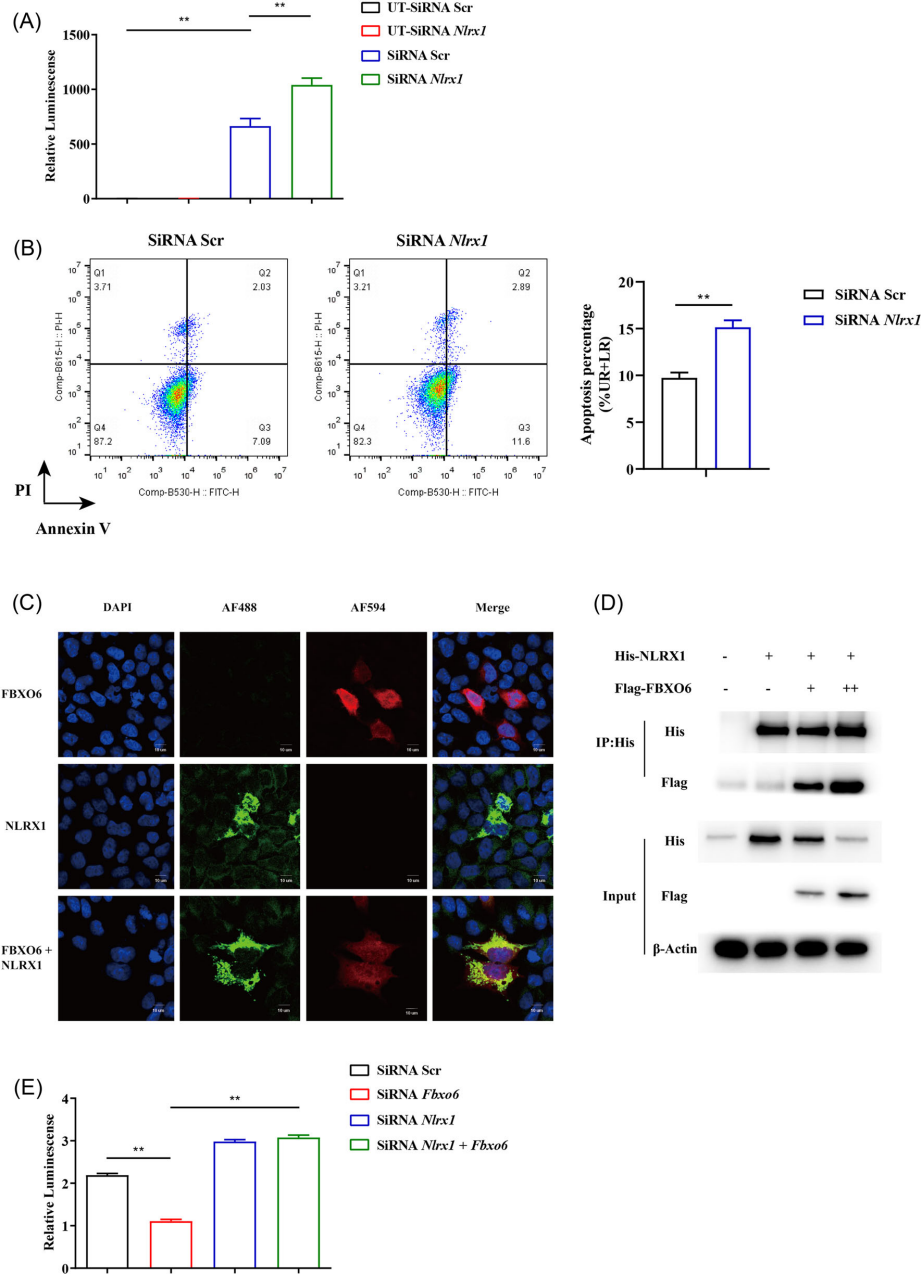
FBXO6 promotes proteasomal degradation of NLRX1. (A) MH‐S cells were transfected with *Nlrx1* or scramble siRNA for 24 h and then infected with PR8‐Renilla (MOI = 1) for 24 h. Then luminescence was measured. (B) MH‐S cells were transfected with NLRX1 or scramble siRNA for 24 h and then infected with PR8 (MOI = 1) for 24 h. The apoptosis of MH‐S cells was measured by flow cytometry. (C) Confocal microscopy of FBXO6 and NLRX1 in HEK293T cells. The cells were transfected with Flag‐FBXO6, His‐NLRX1, or both plasmids for 24 h, followed by staining for anti‐Flag (red) and anti‐His antibody (green) (magnification, ×1200). (D) HEK293T cells were transfected with His‐NLRX1 plasmid along with an increasing amount of Flag‐FBXO6 plasmid (0, 0, 1, or 2 μg). Co‐IP and Western blot analysis of the cell lysates were shown. (E) MH‐S cells were transfected with *Fbxo6, Nlrx1* or scramble siRNA for 24 h and then infected with PR8‐Renilla (MOI = 1) for 24 h. Then luminescence was measured. Quantitative data were presented as mean ± SEM, *p < 0.05; **p < 0.01. MOI, multiplicity of infection; siRNA, small interfering RNA.

NLRX1 was reported to be involved in the apoptosis of macrophages induced by IAV.^19^ Jaworska et al.^19^ reported that NLRX1 did not regulate type I IFN signaling directly during IAV infection but regulated the mitochondrial‐dependent pathway of cell death by binding to one of the proapoptotic IAV protein, namely PB1‐F2. Therefore, we assessed whether the effect of FBXO6 was associated to NLRX1. Firstly, we infected the control and NLRX1 knockdown MH‐S with PR8‐Renilla and measured luminescence in both groups. Consistent with the previous study, NLRX1 knockdown MH‐S exerted worse control of IAV infection (Figure 6A). Simultaneously, increased cell apoptosis in NLRX1 knockdown MH‐S was detected (Figure 6B). Then co‐localization of NLRX1 and FBXO6 was detected by means of immunofluorescence double‐staining and found that the two proteins were well co‐localized in the cytosol when HEK293T cells ectopically overexpressed both proteins (Figure 6C). To further validate the interaction between the NLRX1 and FBXO6, we conducted Co‐IP. NLRX1 could successfully pull down FBXO6 and the input protein level of NLRX1 was downregulated as the FBXO6 increased (Figure 6D). Furthermore, compared to the control group, increased replication of the virus in NLRX1 knockdown MH‐S but no significant difference was observed when MH‐S was knocked down with both NLRX1 and FBXO6 siRNA (Figure 6E). In general, FBXO6 could conform interaction and was responsible for the reduced expression of NLRX1.

### 3.8 FBXO6 promotes K48‐linked ubiquitination and proteasomal degradation of NLRX1

**Figure 7:**
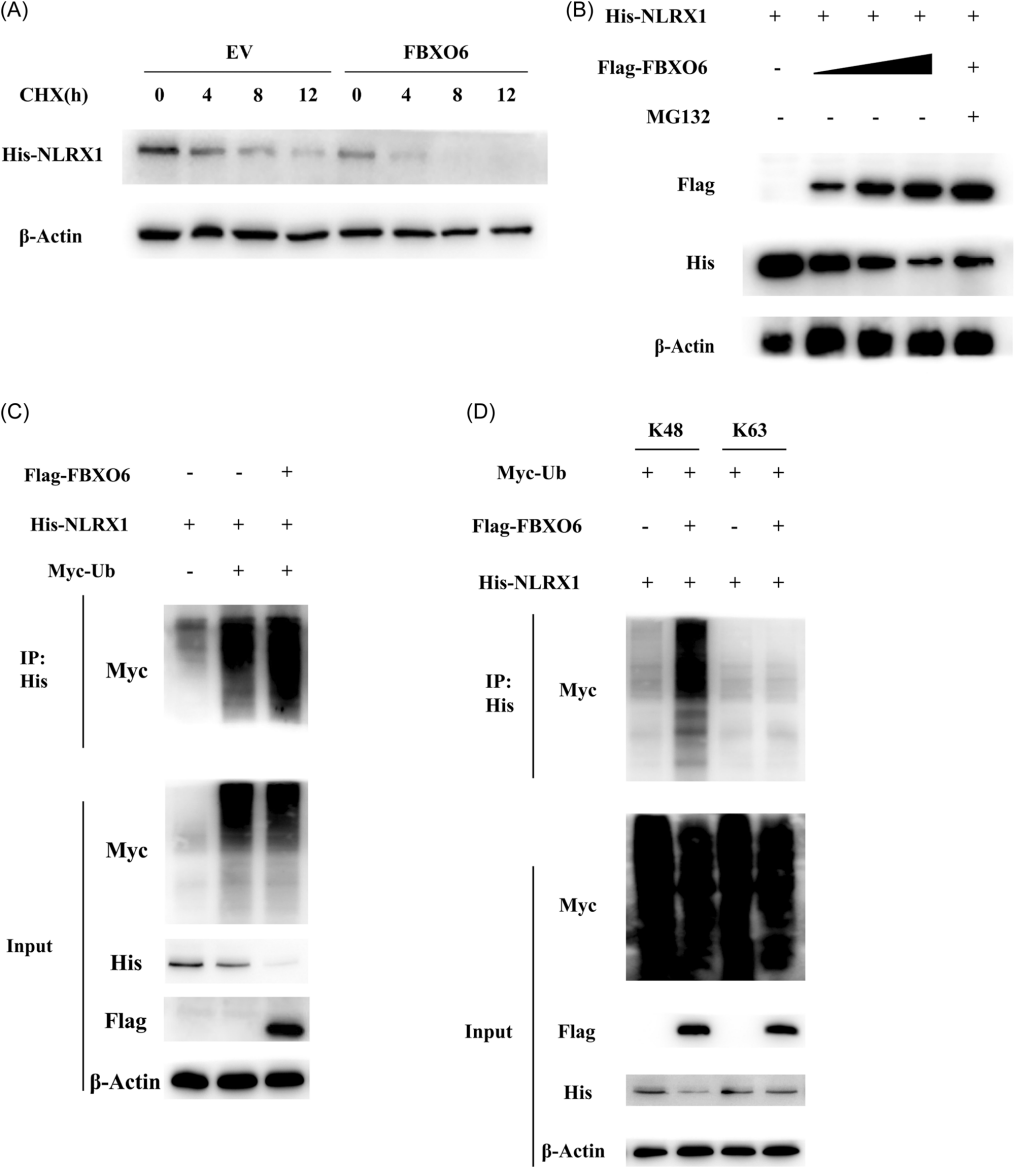
FBXO6 promotes K48‐linked ubiquitination of NLRX1. (A) HEK293T cells were transiently expressing His‐NLRX1 and Flag‐FBXO6 or vector plasmids for 24 h, and then incubated with 10 μM cycloheximide (CHX) for various times. Western blot analysis of cell lysates was shown. (B) HEK293T cells were transfected with His‐NLRX1 plasmid along with an increasing amount of Flag‐FBXO6 plasmid (0, 0.5, 1, or 2 μg), and then treated with MG132 (10 μM) for 4 h. Western blot analysis of the cell lysates were shown. (C) HEK293T cells were transiently co‐transfected with Myc‐Ub, Flag‐FBXO6, along with His‐NLRX1 plasmids. Cell lysates were subjected to immunoprecipitation with anti‐His antibody and immunoblot with anti‐Myc. (D) HEK293T cells were transiently co‐transfected with various combinations of plasmid including Myc‐tagged K48‐linked or K63‐linked ubiquitin, Flag‐FBXO6, and His‐NLRX1. Co‐immunoprecipitation and immunoblot analysis of cell extracts was shown.

To determine the exact approach how FBXO6 influence the protein level of NLRX1. We ectopically expressed His‐tagged NLRX1 and Flag‐tagged FBXO6 in HEK293T cells and treated the cells with the translational inhibitor CHX, and found that CHX did not eliminate the difference between the two groups (Figure 7A). And then we treated HEK293T cells with proteasome inhibitor MG132. FBXO6 mediated downregulation of NLRX1 expression was quantity dependent and could be obviously reversed by MG132 (Figure 7B), but not by CHX (Figure 7A). These results suggested that FBXO6 predominantly functioned during the proteasomal degradation process of NLRX1. Furthermore, Co‐IP analysis revealed that FBXO6 significantly enhanced the polyubiquitination of NLRX1 (Figure 7C). To extend these findings, we attempted to determine the exact type of poly‐ubiquitination linkages involved in NLRX1 degradation. Since K48 and K63 are the two most common linkages reported, we then evaluated the interaction between K48 or K63 and NLRX1 with or without FBXO6 overexpression by using a Co‐IP approach, where we found that FBXO6 noticeably increased the K48‐linked polyubiquitination of NLRX1 (Figure 7D). To sum up, FBXO6 upregulated the proteasomal degradation of NLRX1 through a K48‐linked polyubiquitination manner.

## 4 DISCUSSION

It is well accepted that the innate immune response plays a critical role for the detection of IAV infection, restriction of virus replication, and subsequent activation of adaptive immunity.^37^ Correspondingly, to replicate efficiently in the lungs and rapidly spread to other hosts, IAV have evolved strategies to confront the host defense mechanisms.^38^ During infection, IAV virions initially replicate in pulmonary epithelial cells and are subsequently released into the airspace where they encounter resident AMs. Upon recognizing the viral particles through PRR, AMs will convert into its highly active form and make pro‐inflammatory responses including producing massive inflammatory cytokines/chemokines, namely type I IFNs and downstream ISGs, to restrict early viral replication.^14^ These signals orchestrate the recruitment of other leukocytes to mount a potent antiviral immune response in the lungs.^39^ Therefore, IAV preferentially triggers early apoptosis of macrophages via PB1‐F2 to subvert their function and replicates more efficiently.^19^ We demonstrate here that FBXO6 exerts negatively function in innate antiviral response via promoting proteasomal degradation of NLRX1 to induce the apoptosis of pulmonary resident AMs.

There are few studies regarding the role of FBXO6 in IAV‐induced pneumonia at present. In a recent study of asthma in children, differentially expressed genes screen was conducted in the nasal‐epithelium tissue samples, PB samples, and PB mononuclear cells samples. *Fbxo6* was identified as a hub gene and served an important role in the process of asthma.^40^ By using global *Fbxo6^−/−^* mice, we showed that the deficiency of FBXO6 positively regulated the secretion of type I IFN and ISGs, attenuated viral load in lungs, and facilitated the host survival. AMs have been reported to play a critical role in host survival upon influenza virus infection by reducing lung function decline and thereby protecting the host from asphyxiation. Specifically, at the early stage of viral infection, especially in the first 3 days postinfection, AMs act as the major player in restricting viral replication and spread.^14^, ^41^, ^42^ Thus, we sought to measure the frequency of macrophages at 3 dpi, when the viral replication peaks according to previous studies.^19^ As a result, the amount of AMs was significantly increased in FBXO6 deficient mice. Then we wanted to investigate whether cell proliferation or death plays the dominant part in this progress. Considering apoptosis was the most common pathway of cell death, we carried out the proliferation and apoptosis detection of AMs by flow cytometer. The proliferation of AMs did not show a statistical difference while the apoptosis of AMs was significantly reduced in mice with FBXO6 deficiency.

In our in vitro analysis, consistent with in vivo results, partial silence of FBXO6 by siRNA inhibited the apoptosis of MH‐S, enhanced the expression of IFN‐β and ISGs, and restricted the viral replication compared to the control. Through a literature search, we focused on NLRX1, a PRR classified to the NLR family, which was identified to have the capacity to bind PB1‐F2 and defend immune cells from virus‐induced apoptosis.^19^ Our results elucidated FBXO6 had interaction with and facilitated the proteasomal degradation of NLRX1. In a previous report, NLRX1 deficient macrophages presented a higher apoptosis rate after IAV infection both in vivo and in vitro.^19^ Consistently, the knockdown of NLRX1 indeed promoted the apoptosis frequency of MH‐S. Still, there remain controversies whether NLRX1 influences RIG‐I/MAVS‐dependent signaling pathway^43^, ^44^, ^45^ but evidence constantly suggests that NLRX1‐deficiency does not affect the mortality of IAV‐infected mice.^43^ Nevertheless, in our study FBXO6 deficient mice presented a higher survival rate than WT mice. In our previous study, we reported FBXO6 interacts with IRF3 via its FBA domain and thus negatively regulates the homeostasis of IFN‐I production.^32^ Thus, we speculate FBXO6 may possess multiple functions in IAV infection. Considering the potent influence of FBXO6 in regulating the activation of IFN‐I signaling by targeting the key transcription factor IRF3,^32^ we then utilized a broad caspase inhibitor named emricasan to block the apoptosis of MH‐S. Interestingly, appreciable differences in FBXO6 knockdown group with or without emricasan treatment were observed, which revealed FBXO6‐deficiency mediated cell protection against AMs apoptosis is indispensable in antiviral immunity.

**Figure 8:**
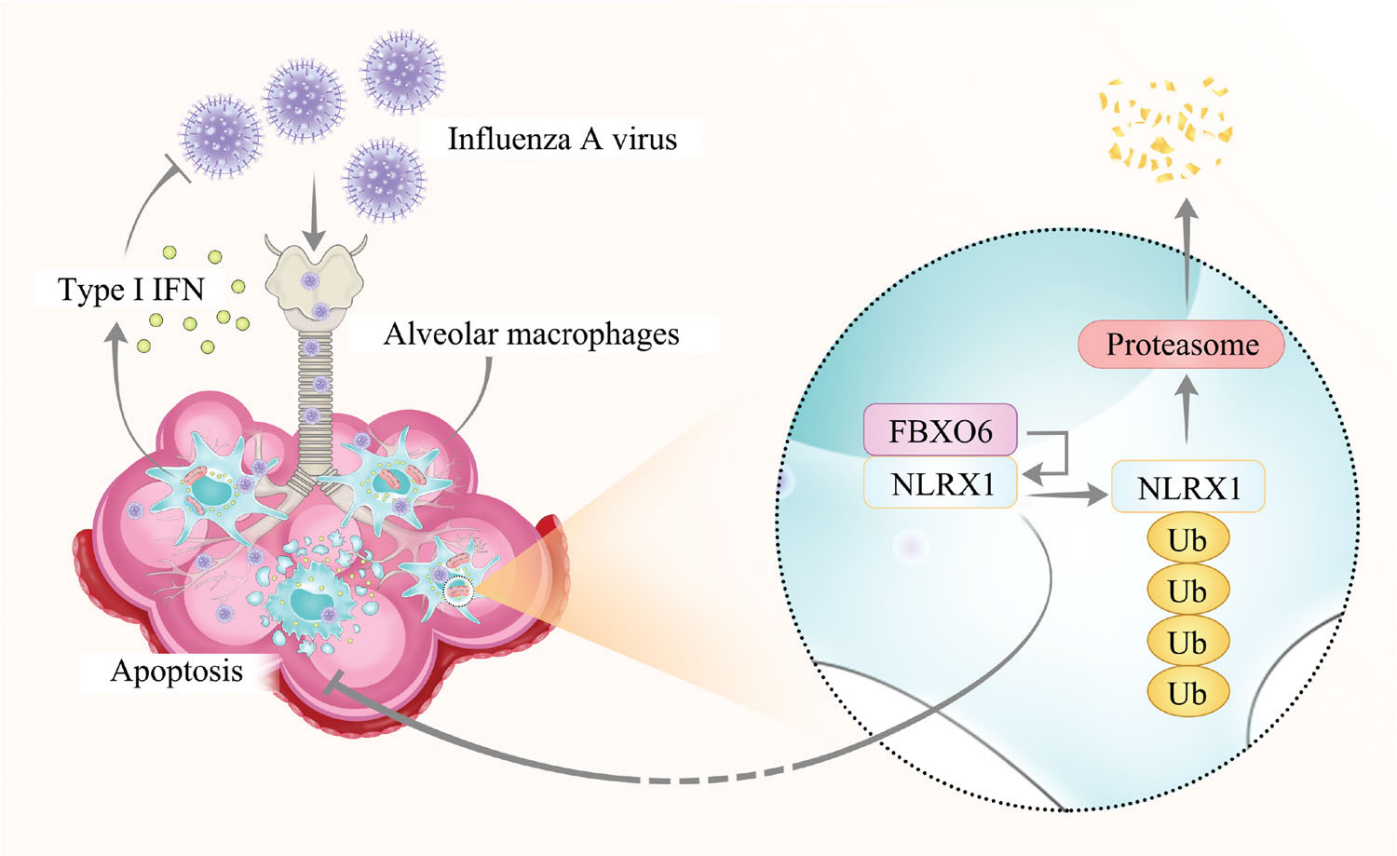
Possible molecular mechanisms of FBXO6 negatively regulating antiviral immunity of the host. Upon infection with IAV,   FBXO6 promotes proteasomal degradation of NLRX1, thus enhances alveolar macrophages apoptosis and impairs antiviral immunity of the host. IAV, influenza A virus.

In conclusion, the results  here demonstrate that FBXO6 regulates AMs apoptosis by influencing the proteasomal degradation of NLRX1, thus impairs the survival of macrophages and antiviral immunity of the host infected with IAV (Figure 8).

## AUTHOR CONTRIBUTIONS

Mengyuan Cen, Wei Ouyang, Xiuhui Lin, Xiaohong Du, Huiqun Hu, Huidan Lu, Wanying Zhang, and Jingyan Xia performed the experiments and analyzed the data. Mengyuan Cen, Wei Ouyang, Xiaofeng Qin, and Feng Xu prepared and revised the manuscript. Feng Xu and Xiaofeng Qin originated the hypothesis and designed the research. All authors read and approved the content of the manuscript.

## CONFLICT OF INTEREST

The authors declare no conflict of interest.

## Supporting information

Supplementary information.

## Data Availability

The data that support the findings of this study are available from the corresponding author upon reasonable request. All data in our study are available upon request.
