# Supplementary material for: FBXO6 regulates the antiviral immune responses via mediating alveolar macrophages survival
Source: J Med Virol. 2022 Oct 31;95(1):e28203. doi: 10.1002/jmv.28203 (PMC10092588; doi:10.1002/jmv.28203)
Supplement: Supplementary file 1 — Supplementary information. [file JMV-95-0-s001.docx]

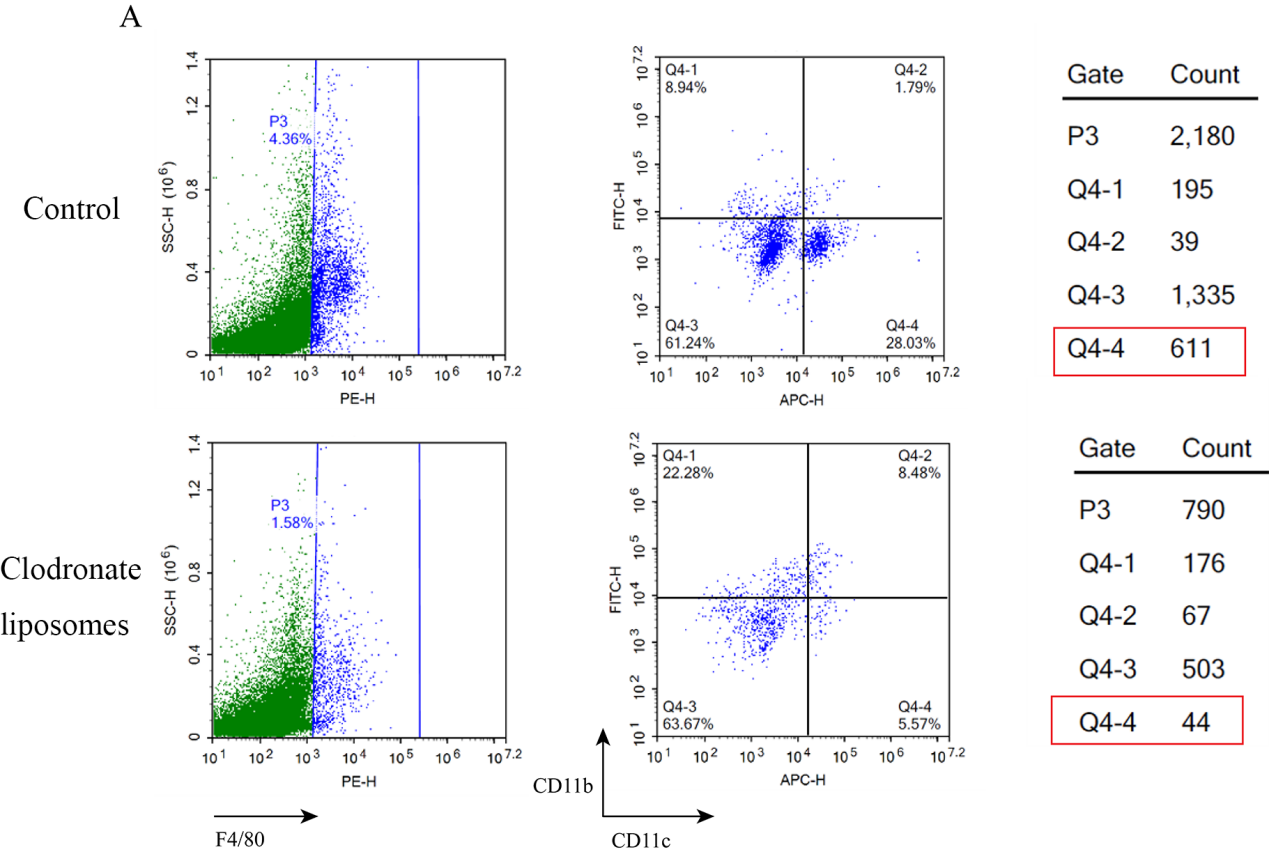


Fig S1: (A) WT mice were treated with 50μl clodronate liposomes for 2 days and then their lungs were collected and analyzed by flow cytometry.

The sequences of primers for qPCR are as follows:

*β-actin* forward, 5’-GTATCCTGACCCTGAAGTACC-3’;

and reverse, 5’-GAAGGTCTCAAACATGATCT-3’;

*Ifn-α* forward, 5’-TACTCAGCAGACCTTGAACCT-3’;

and reverse, 5’-CAGTCTTGGCAGCAAGTTGAC-3’;

*Ifn-β* forward, 5’- ATGAGTGGTGGTTGCAGGC -3’;

and reverse, 5’- TGACCTTTCAAATGCAGTAGATTCA -3’;

*Ifit1* forward, 5’- CAAGGCAGGTTTCTGAGGAG -3’;

and reverse, 5’- GACCTGGTCACCATCAGCAT -3’;

*Ifit3* forward, 5’- TTCCCAGCAGCACAGAAAC -3’;

and reverse, 5’- AAATTCCAGGTGAAATGGCA -3’;

*Ccl5* forward, 5’- GCTGCTTTGCCTACCTCTCC-3’;

and reverse, 5’-TCGAGTGACAAACACGACTGC-3’;

*Cxcl10* forward, 5’- CCTGCCCACGTGTTGAGAT -3’;

and reverse, 5’- TGATGGTCTTAGATTCCGGATTC -3’;

*Mx1* forward, 5’- GTGGTAGTCCCCAGCAATGT -3’;

and reverse, 5’-TGCTGACCTCTGCACTTGAC -3’;

*M1* forward, 5’- ATGAGCCTTCTAACCGAGGTCGAAACG -3’;

and reverse, 5’- TGGACAAAACGTCTACGCTGCAG -3’.

The siRNA sequences targeting mouse FBXO6 were as follows:

sense,5’- CCCACACCUUCUCUGAUUATT -3’,

and antisense, 5’- UAAUCAGAGAAGGUGUGGGTT -3’.

The siRNA sequences targeting mouse NLRX1 were:

sense, 5’- GCUUUCUACGCCUGAACUUTT -3’,

and antisense: 5’- AAGUUCAGGCGUAGAAAGCTT -3’.

The scramble control sequences were:

sense, 5’-UUCUCCGAACGUGUCACGUTT-3’,

and antisense: 5’-ACGUGACACGUUCGGAGAATT-3’.
